# The cytotoxicity of coxsackievirus B3 is associated with a blockage of autophagic flux mediated by reduced syntaxin 17 expression

**DOI:** 10.1038/s41419-018-0271-0

**Published:** 2018-02-14

**Authors:** Lang Tian, Yeyi Yang, Chunyun Li, Jia Chen, Zhuoying Li, Xin Li, Shentang Li, Fang Wu, Zhangxue Hu, Zuocheng Yang

**Affiliations:** 1 Department of Pediatrics, The Third Xiangya Hospital, Central South University, 410013 Changsha, China; 2 Department of Medicine, The Third Xiangya Hospital, Central South University, 410013 Changsha, China; 3 Department of Pediatrics, Daping Hospital and Field Surgery Institute, Third Military Medical University, 400042 Chongqing, China

## Abstract

Coxsackievirus B3 (CVB3) is an important human pathogen linked to cardiac arrhythmias and acute heart failure. CVB3 infection has been reported to induce the formation of autophagosomes that support the viral replication in host cells. Interestingly, our study shows that the accumulation of autophagosomes during CVB3 infection is caused by a blockage of autophagosome–lysosome fusion rather than the induction of autophagosome biogenesis. Moreover, CVB3 decreases the transcription and translation of syntaxin 17 (STX17), a SNARE (soluble *N*-ethylmaleimide-sensitive factor activating protein receptor) protein involved in autophagosome–lysosome fusion. Overexpression of STX17 restored the autophagic flux, alleviated the virus-induced lysosomal dysfunction, and decreased the apoptosis induced by CVB3 infection in HeLa cells. Taken together, our results suggest that CVB3 infection impairs the autophagic flux by blocking autophagosome–lysosome fusion. These findings thus point to potential new therapeutic strategies targeting STX17 or autophagosome–lysosome fusion for treating CVB3-associated diseases.

## Introduction

Coxsackievirus B (CVB) belongs to the genus *Enterovirus* in the family Picornaviridae. Symptoms of infection with viruses in the Coxsackie B group include fever, headache, sore throat, and gastrointestinal distress, as well as chest and muscle pain. This presentation is known as epidemic pleurodynia or Bornholm disease. CVB are also the most common cause of infectious myocarditis, which can lead to dilated cardiomyopathy and cardiac failure resulting in permanent heart damage or death^1^. Moreover, this group of viruses contains some of the most common pathogens affecting children and young adults^2–4^, which makes it an important study subject for pediatricians. These viruses are also a frequent cause of pancreatitis and aseptic meningitis^5,6^. Even though they are one of the most common causes of unexpected sudden death, there is no well-accepted treatment for the Coxsackie B3 group of viruses^7^. The study of new therapeutic drugs depends on the continued investigation of the molecular mechanisms of CVB cytotoxicity. As an important member of the CVB group, coxsackievirus B3 (CVB3) is an etiologic agent of many different human infections that range in severity from asymptomatic to lethal. The studies have suggested that CVB3 might cause cell death by interfering with autophagy^8–10^, but the exact mechanism remains unclear.

Autophagy is a complex catabolic process by which cells remove and recycle protein aggregates and damaged organelles^11^. It is indispensable for cellular homeostasis and is involved in many developmental pathways as well as disease processes^12,13^. Autophagy can also serve as an innate immune mechanism to remove intracellular microbial pathogens, in a process that is specific for the removal of viruses and bacteria, termed xenophagy. Autophagy involves the formation of double-membrane vesicles termed autophagosomes, which are delivered to lysosomes for destruction. Recent studies have also suggested that autophagy might act as either an antiviral or a proviral mechanism^14^. Autophagy protects against many infections by inducing the lysosome-mediated degradation of invading pathogens^15^. Nevertheless, although autophagy can suppress the replication of certain viruses, in vitro studies suggest that some enteroviruses not only evade these protective mechanisms, but actually hijack autophagy to facilitate their own replication^16^. CVB3 also appears to increase the abundance of these small vesicles by inducing autophagy^9^. The autophagy marker LC3-II (lipidated microtubule-associated protein 1 light chain 3) was significantly increased in both the myocardium and cardiac myocytes extracted from the ventricles of mice infected with CVB3^17^. It has been demonstrated that CVB3 replication is supported by the assembly of autophagosomes^18^. Moreover, the expression of protein 2B, which is a CVB3 protein containing an autophagy-inducing motif in the region 36–83 amino acids, was sufficient to induce autophagy by itself^19,20^. However, the molecular mechanism by which CVB3 manipulates autophagy is poorly understood.

Here, we report on the CVB3-induced accumulation of autophagosomes, which appears to be mediated by the inhibition of autophagosome–lysosome fusion. Moreover, we demonstrated that the transcription of the SNARE (soluble *N*-ethylmaleimide-sensitive factor activating protein receptor) protein syntaxin 17 (STX17) was decreased in CVB3-infected HeLa cells. Furthermore, STX17 overexpression rescued the CVB3-induced inhibition of autophagosome–lysosome fusion and alleviated CVB3-induced apoptosis, proving that its downregulation is a necessary factor of CVB3’s interference with autophagy.

## Results

### CVB3 infection increases autophagosome formation

**Fig. 1:**
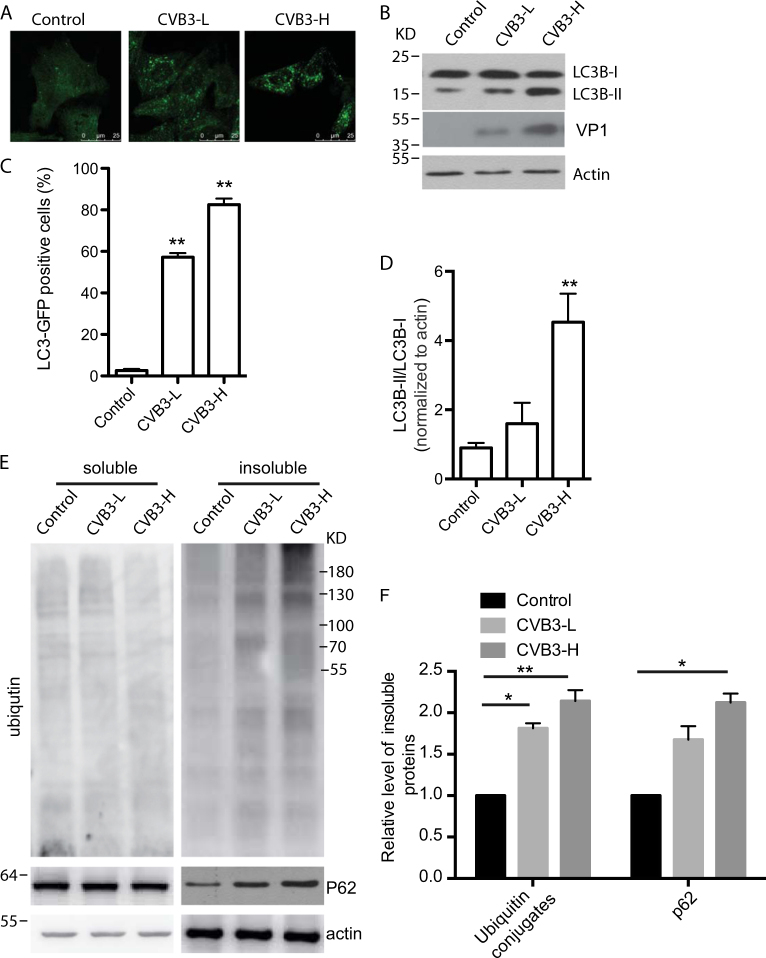
CVB3 induces LC3-II accumulation in dose-dependent manner. **a** HeLa cells were infected with CVB3 at a low (CVB3-L) and high MOI (CVB-H) and incubated for 36 h. Endogenous LC3B was detected by immunofluorescence. **b** Western blotting was employed to detect LC3B; VP1: a CVB3 viral protein; p62: a substrate for autophagic degradation; actin: a loading control. **c** Statistical analysis for **a** vs. Control, **p < 0.01. **d** Statistical analysis of the ratio of LC3B-II to LC3B-I, normalized to actin for **b** vs. Control, **p < 0.01. **d** Western blotting was employed to detect Triton X-100-soluble and Triton X-insoluble ubiquitin-conjugated proteins after infection with CVB3 at low (CVB3-L) and high MOI (CVB-H), respectively, and incubation for 36 h. Actin is a loading control. **e** Statistical analysis of the ratio of insoluble ubiquitin conjugates to actin, normalized to actin for **d**. *p < 0.05, **p < 0.01

Punctate accumulation of LC3, which indicates the recruitment of LC3 to autophagic vacuoles, has been suggested to be a hallmark of autophagy^21^. Thus, to determine whether CVB3 infection triggers cellular autophagy, endogenous LC3 was detected by immunofluorescence. After CVB3 infection of HeLa cells at low and high multiplicity of infection (MOI), we observed a marked punctate LC3 distribution, which increased in a dose-dependent manner (Fig. 1a, c). To further confirm the increased formation of autophagosomes after viral infection, we examined LC3 modification, another hallmark of autophagy^22^. Two forms of LC3 have been reported. In quiescent cells, LC3 resides in the cytoplasm in a precursor form, referred to as LC3-I. However, upon exposure to various autophagic stimuli, it is rapidly degraded and subsequently conjugated to phosphatidylethanolamine to form an LC3–phosphatidylethanolamine complex, commonly referred as LC3-II, which participates in autophagosome formation and remains attached to mature autophagosomes. Moreover, it has been shown that the LC3-II/LC3-I or LC3-II/actin ratio correlates well with the number of autophagosomes^21,22^. Therefore, western blots were performed after CVB3 infection to detect the LC3-II/LC3-I ratio. As shown in Fig. 1b, d, the concentration of LC3-II increased with the progression of CVB3 infection, which was accompanied by a decrease of LC3-I expression. The densitometric ratio of LC3-II to LC3-I demonstrated a cumulative increase of autophagosomes during CVB3 infection. However, we did not observe a change of Triton X-100-soluble p62 (Fig. 1e), a protein marker of autophagy-mediated protein degradation, that is, autophagic flux. Similar results were found during CVB3 infection in HEK293 cells^18^. However, Triton X-100-insoluble proteins were detected and showed a significant increase of both ubiquitin conjugates and p62, indicating a dysfunction of autophagic–lysosomal degradation (Fig. 1e, f) ^23^.

### CVB3 infection blocked the autophagic flux

**Fig. 2:**
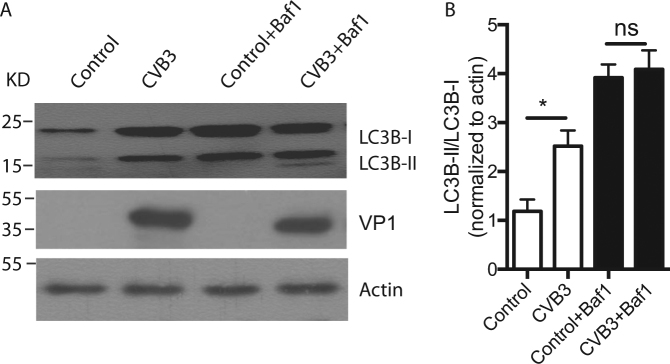
Baf1 blocks accumulation of LC3-II induced by CVB3 infection. **a** HeLa cells were infected with CVB3 at a high MOI and treated with 50 nM Baf1 for 12 h. Endogenous LC3B was detected by western blotting. VP1: a CVB3 viral protein. Actin: a loading control. **b** Statistical analysis of the ratio of LC3B-II to LC3B-I, normalized to actin vs. Control. *p < 0.01. NS, no statistical significance

One of the principal methods currently used to measure autophagic flux is the monitoring of LC3 turnover, which is based on the observation that LC3-II is degraded in autolysosomes^22^. If cells are treated with lysosomotropic reagents such as ammonium chloride, chloroquine, or bafilomycin A1 (Baf1), which inhibit the acidification inside the lysosomes or autophagosome–lysosome fusion, or with inhibitors of lysosomal proteases such as E64d and pepstatin A, the degradation of LC3-II is blocked, resulting in the accumulation of LC3-II^24^. Accordingly, the differences in the amounts of LC3-II between samples in the presence and absence of lysosomal inhibitors represent the amount of LC3 that is delivered to lysosomes for degradation (i.e., autophagic flux). Compared to CVB3-infected cells, our results showed little increase in LC3-II accumulation when cells were treated with both the virus and Baf1 (Fig. 2a, b). This implies that the CVB3-induced accumulation of LC3-II results from a blockage of autophagic flux, and not from an activation of autophagy.

**Fig. 3:**
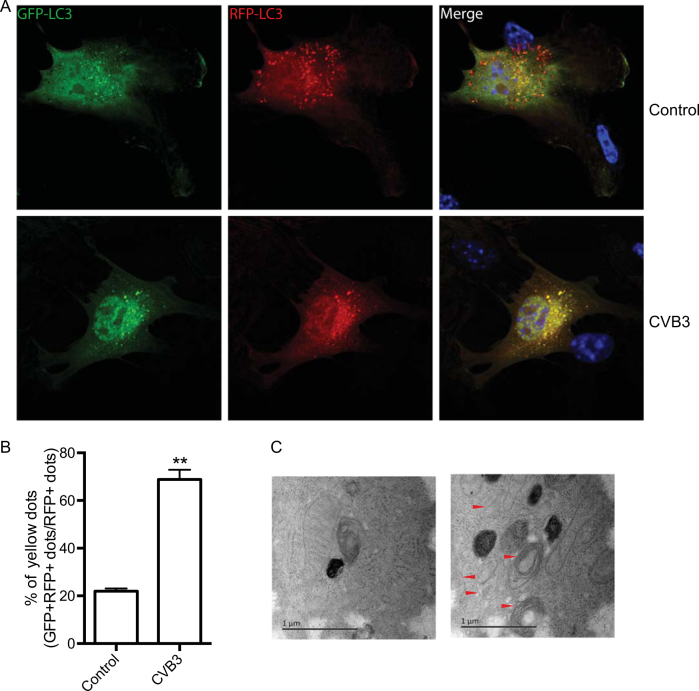
CVB3 infection increases autophagic vesicles by blockage of autophagosome–lysosome fusion. **a** The monomeric red fluorescent protein (mRFP)-GFP-LC3 tandem reporter was used to transfect HeLa cells with or without CVB3 infection. If autophagic flux is increased, the number of both yellow and red puncta are increased; if autophagosome maturation into autolysosomes is blocked, only the number of yellow puncta is increased, without a concomitant increase of the number of red puncta. **b** After 24 h of transfection, yellow puncta were counted. Quantification of the percentage of yellow dots (GFP+/RFP+ dots), which contain both GFP and mRFP signal (autophagosomes), out of the total number of red dots (RFP+ dots) vs. Control. **p < 0.01. **c** A representative ultrastructural picture of CVB3-infected HeLa cells. Autophagosome-like vacuoles are indicated by arrows. Scale bar = 1 µm

To further investigate the mechanisms by which CVB3 impairs autophagic degradation, we used HeLa cells expressing a monomeric red fluorescent protein (mRFP)-GFP-LC3 tandem reporter. GFP fluorescence is rapidly quenched in the acidic lysosomal environment, whereas mRFP fluorescence persists^25^. In control cells, we observed the accumulation of many red puncta, alongside a smaller number of yellow puncta, suggesting a normal autophagic flux. By contrast, CVB3 infection led to a massive accumulation of yellow puncta, suggesting an impaired transition of GFP and mRFP double-positive autophagosomes to mRFP-positive, GFP-negative autolysosomes (Fig. 3a, b). This could be due to either a decrease in autophagosome–lysosome fusion or an increase of lysosomal pH. To discriminate between these two possibilities, ultrastructural analysis was performed on sham-infected or CVB3-infected HeLa cells. Electron micrographs revealed a much higher accumulation of double-membrane vesicles in CVB3-infected cells than in the sham-infected controls (Fig. 3c), suggesting that the fusion of autophagosomes and lysosomes was blocked.

### Transcription of STX17 was decreased upon CVB3 infection

**Fig. 4:**
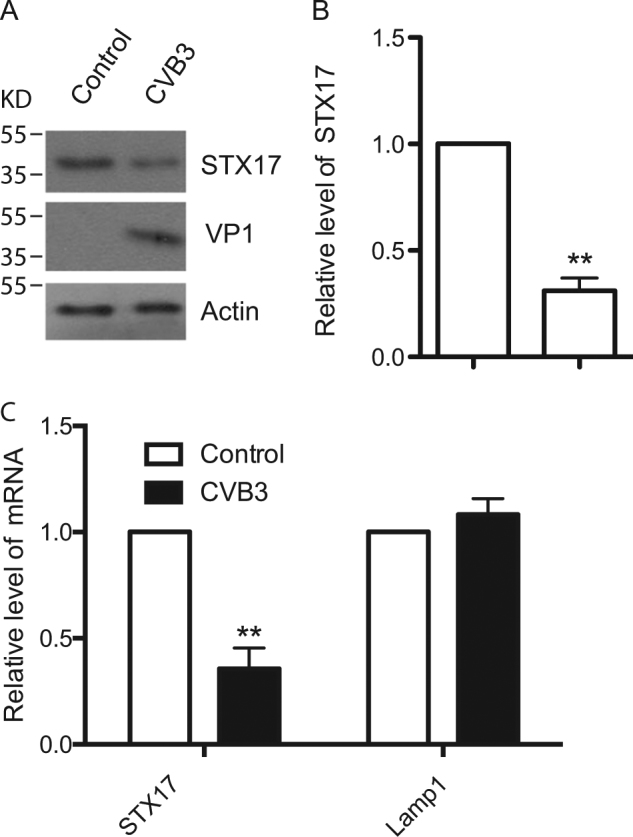
CVB3 infection inhibits transcription of STX17. **a** Endogenous STX17 was detected in CVB3-infected HeLa cells by western blotting. VP1: a CVB3 viral protein. Actin: a loading control. **b** Statistical analysis for **a** vs. Control. **p < 0.01. **c** Transcription of STX17 and Lamp1 was measured in CVB3- infected HeLa cells by real-time PCR vs. Control. **p < 0.01

To further investigate the mechanisms by which CVB3 impaired the autophagosome–lysosome fusion, we tested whether the expression of the autophagosomal SNARE-mediated autophagosome–lysosome fusion protein syntaxin 17 (STX17) was changed in the CVB3-infected cells. Western blot analysis revealed a significant decrease of STX17 protein levels in CVB3-infected cells (Fig. 4a, b). To determine whether the CVB3 infection had an effect on the transcription of STX17, real-time PCR was performed. Our results showed that CVB3 specifically downregulated the expression of STX17. Moreover, an unrelated transcription control—the expression of the lysosomal protein Lamp1, was unchanged during CVB3 infection (Fig. 4c)

### Overexpression of STX17 rescued the accumulation of autophagosomes induced by CVB3 infection

**Fig. 5:**
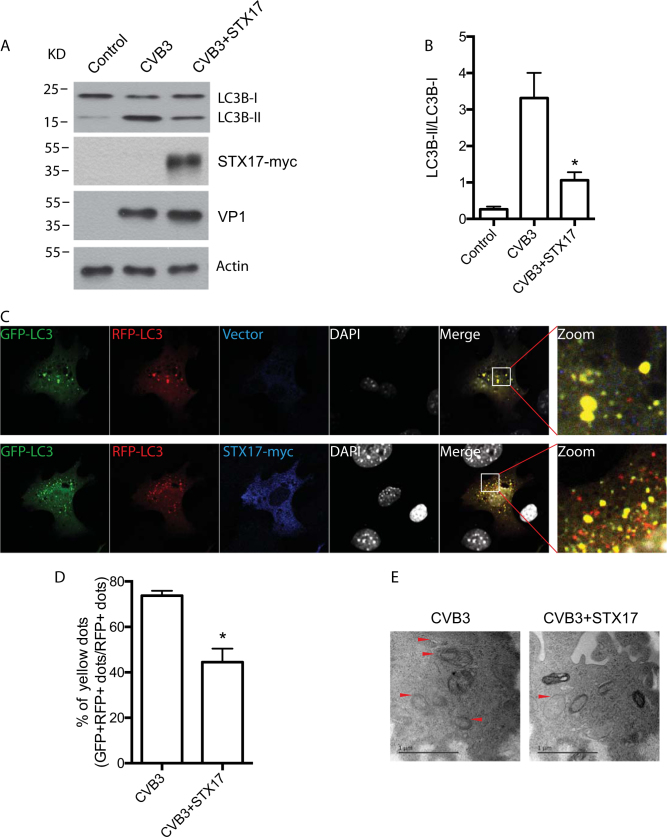
Exogenous STX17 restores impaired autophagosome–lysosome fusion following CVB3 infection. **a** CVB3-infected HeLa cells were transfected with STX17-myc or empty vector. LC3 was detected in CVB3-infected HeLa cells by western blotting. VP1: a CVB3 viral protein. Actin: a loading control. **b** Statistical analysis for **a** vs. CVB3. *p < 0.05. **c** The monomeric red fluorescent protein (mRFP)-GFP-LC3 tandem reporter and STX17-myc were used to transfect HeLa cells with CVB3 infection. **d** Yellow puncta were counted. Quantification of the percentage of yellow dots (GFP+/RFP+ dots), which contain both GFP and mRFP signal (autophagosomes), out of the total number of red dots (RFP+ dots). vs. CVB3. *p < 0.05. **e** Ultrastructural analysis of CVB3-infected HeLa cells transfected with STX17-myc or sham. Autophagosome-like vacuoles are indicated by arrows. Scale bar = 1 µm

To further confirm the blockage of autophagic flux caused by CVB3, STX17 was overexpressed in HeLa cells infected with CVB3. The results showed that STX17 alleviated the accumulation of LC3-II induced by CVB3 infection (Fig. 5a, b). The accumulation of autophagosomes induced by CVB3 infection was also reduced when STX17 expression was reintroduced. The accumulation of yellow puncta in the mRFP-GFP-LC3 tandem reporter system was decreased in STX17-transfected cells compared with the controls only infected with CVB3, suggesting that mRFP-positive, GFP-negative autolysosomes were rescued by STX17 (Fig. 5c, d). Electron micrographs of the STX17-overexpressing cells also demonstrated a decrease in the number of autophagosome-like vesicles, which are defined as double-membrane vesicles present in the cytoplasm, indicating an increased formation of autophagosomes in the cells infected only with CVB3 (Fig. 5e).

### Ectopic expression of STX17 restored normal lysosome function and protected from CVB3-induced cell death

**Fig. 6:**
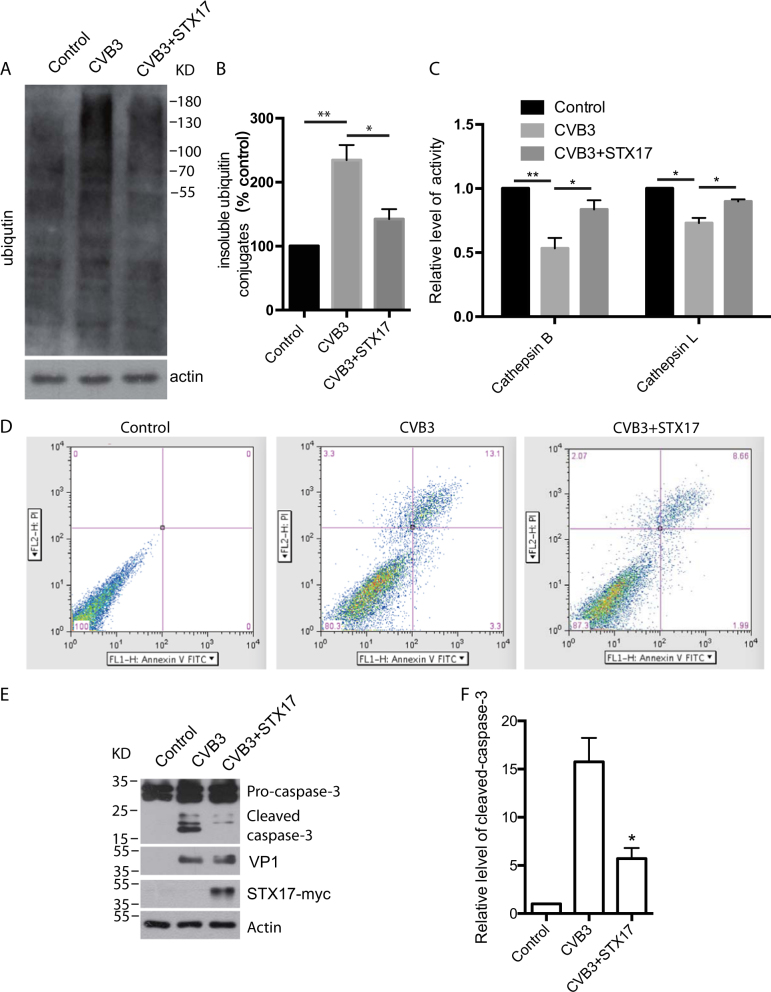
STX17 restored lysosomal function and blocked apoptosis induced by CVB3 infection. **a** Western blotting was employed to detect Triton X-100-insoluble ubiquitin-conjugated proteins in cells with or without exogenous expression of STX17 after infection with CVB3. **b** Statistical analysis of the ratio of insoluble ubiquitin conjugates to actin, normalized to actin for **a**, *p < 0.05, **p < 0.01. **c** The activity of cathepsin B/L was measured in cells with or without exogenous expression of STX17 after infection with CVB3, *p < 0.05. **d** Annexin V + PI staining for the flow-cytometric measurement of apoptosis induced by CVB3 infection. **e** Cleaved caspase-3 was detected in CVB3-infected HeLa cells transfected with STX17-myc or sham. **f** Statistical analysis for **a** vs. CVB3. *p < 0.01

Activation of lysosome function depends on autophagosome–lysosome fusion, and blockage of fusion effectively decreases cathepsin B activity^26^. The reduced lysosome degradation capacity can be detected by measuring the presence of Triton X-100-insoluble ubiquitinated proteins and protease activity^27^. CVB3 infection markedly increased the amount of Triton X-100-insoluble ubiquitin-conjugated proteins (Fig. 6a, b), and reduced the activity of cathepsin B/L (Fig. 6c). Moreover, these changes were rescued by exogenous STX17. Flow-cytometric analysis revealed that the percentage of annexin V-positive (apoptotic) cells was increased by CVB3 infection. Our data also indicated that STX17 overexpression significantly blocked the CVB3-induced apoptosis in HeLa cells (Fig. 6d). Apoptosis-related caspase molecules were activated by cleavage in a time-dependent manner after CVB3 infection, suggesting that CVB3-induced apoptosis was partially dependent on the caspase pathway^28–31^. To confirm the protective effect of STX17 against cell death induced by CVB3 infection, cleaved caspase-3 was detected by western blot analysis, and the results showed that STX17 indeed inhibited the cleavage of caspase-3 in CVB3-infected HeLa cells (Fig. 6e, f).

## Discussion

In line with several earlier reports, we found that CVB3 induced autophagosome accumulation in HeLa cells^9,17,20,28,32,33^. Wong et al. ^18^ were the first to show via ultrastructural microscopy that the number of double-membrane vesicles increased dramatically following CVB3 infection of both HeLa and HEK293 cells^18^. Moreover, it was reported that the autophagosome supports CVB3 replication^18^. Similarly, the autophagosome provides an intracellular compartment that benefits the survival and multiplication of some bacteria, including *Helicobacter pylori*^34^, *Porphyromonas gingivalis*, *Brucella abortus*, and *Coxiella burnetii*^35^. Conversely, inhibition of autophagosome formation reduced the replication of CVB3^18^. Autophagy activation is expected to result in an increase in the number of all autophagic structures (i.e., isolated membranes, autophagosomes, and autophago-lysosomes). However, protein expression of p62, a marker of autophagy-mediated protein degradation or autophagic flux, was not decreased during the course of CVB3 infection, suggesting that CVB3 infection triggers increased accumulation of autophagosomes without promoting autophagic flux. It was reported that the level of p62 does not correlate with autophagy following CVB3 infection, due to cleavage of the protein by viral protease 2A^pro^^36^, or caspases and calpains^22^. It is well known that ubiquitin-positive aggregates and p62 accumulate in the tissues of autophagy-related knockout mice^23,37^. Subsequently, we determined the amounts of Triton X-100-soluble and insoluble ubiquitin conjugates after infection with CVB3. A dose-dependent increase of Triton X-100-insoluble ubiquitin conjugates and p62 indicated a blockage of autophagic flux in CVB3-infected cells. If any step upstream of autophagosome formation is blocked, the number of autophagic structures will be decreased. By contrast, a blockade of autophagosome–lysosome fusion or degradation increases the number of autophagosomes. Our data therefore clearly indicate that the CVB3-induced accumulation of autophagosomes is due to a blockage of autophagosome–lysosome fusion.

Autophagosome formation requires extensive membrane remodeling, which is also induced during the replication of positive-strand RNA viruses. Indeed, many positive-strand RNA viruses, including picornaviruses and flaviviruses, induce the autophagic process during their replicative life cycles to generate the membranes necessary for the biogenesis of their replication organelles^38^. In addition, a diverse array of other viruses also induce autophagy, including members of the Paramyxoviridae, Orthomyxoviridae, Togaviridae, and Herpesviridae^39^. Interestingly, several herpesviruses express proteins that directly inhibit the formation of autophagosomes, indicating that these viruses may have evolved strategies to evade the degradative nature of the autophagic pathway^39^. Although we observed that CVB3 induced the accumulation of autophagosomes, the autophagic flux was actually blocked via impaired autophagosome–lysosome fusion. Therefore, degradative autophagy was inhibited. Importantly, we found that CVB3 suppressed the transcription of the SNARE protein STX17, which interacts with the late endosome/lysosome membrane proteins VAMP8 and SNAP29 to promote the fusion of autophagosomes with lysosomes^40^. Depletion of STX17 causes the accumulation of autophagosomes with impaired degradation capacity^41^. A recent report has shown that infection by human *Parainfluenza virus* type 3 induces the accumulation of cytoplasmic autophagosomes via a direct blockage of autophagic flux, whereby a viral phosphoprotein binds SNAP29 and inhibits its interaction with STX17, thereby preventing these two host SNARE proteins from mediating autophagosome–lysosome fusion^42^. In agreement with our results, it was found that HCV-replicating cells have a decreased amount of STX17 due to its impaired transcription and rapid turnover^43^. Moreover, exogenous STX17 diminished the number of released infectious viral particles and decreases the amount of retained intracellular viral particles by promoting autophagosome–lysosome fusion and degradation of viral particles in HCV-replicating cells^43^.

Consistently with a potential mechanism of downregulated transcription of STX17, it has been proved that picornavirus proteases 2A and 3C contribute to viral pathogenesis by decreasing host gene expression through cleavage of transcription factors^44^. Similarly, CVB3 protease 3C was found to cleave the inhibitor of κBα (IκBα). After cleavage, a proteolytic fragment of IκBα can form a stable complex with nuclear factor-κB (NF-κB) that translocates to the nucleus and inhibits NF-kB transactivation^45^. In poliovirus infection, protease 3C can cleave and inactivate a number of transcription factors and co-activators, including activator Oct-1, cyclic AMP-responsive element-binding protein, TATA-binding protein, and transcription factor IIIC2^46^.

Increased apoptosis is also correlated with viral replication in the hearts of mice and cells infected with CVB3 in vitro^47^. In fact, viral proteins have been directly linked to apoptosis or the expression of pro-apoptotic proteins during virus infection. Specifically, the pro-apoptotic protein Siva, which binds to the CVB3 capsid protein VP2, is strongly upregulated in the same region where apoptosis occurs during acute CVB3-induced myocarditis^31^. The intrinsic apoptotic pathway may be involved in virus-induced cell death. CVB3 infection was also demonstrated to affect GSK3β^48^ and cyr61^49^, which contributes to CVB3-induced apoptosis. Our results demonstrate that CVB3 infection interferes with the lysosomal degradation function through a blockage of autophagosome–lysosome fusion. The resulting deficiency of autophagy leads to the accumulation of Triton X-100-insoluble ubiquitinated proteins, cell death^50^, and increased generation of reactive oxygen species (ROS)^51,52^. Furthermore, ROS were associated with high mortality and had a role in the pathogenesis of cardiac damage following CVB3 infection^53^. Restoration of autophagic flux by exogenous STX17 expression also may promote the degradation of viral-apoptotic or pro-apoptotic proteins via the autophagy–lysosome pathway to reduce cell death and pathogenesis.

Picornavirus requires autophagic double-membrane vesicles, which promote viral RNA replication and protect newly synthesized viral RNA, for optimal infection and pathogenesis^33,54,55^. We found that the accumulation of autophagic vesicles was induced by CVB3 infection, that the autophagic flux was blocked at the autophagosome–lysosome fusion step, and that this blockage was mediated by reduced STX17 expression. Although overexpression STX17 have shown a declining trend, it did not significantly alter CVB3 replication by detection of VP1 protein in our system. It is reported that autophagy could be induced by rapamycin without altering levels of CVB3 replication in neural progenitor and stem cells with a higher basal levels of autophagy. However, the autophagosome is essential for CVB3 replication^10^. Our result also shows that 3-MA dramatically reduces the CVB3 replication by inhibiting autophagosome formation (data not shown). We speculate that the lower level of autophagy modulate CVB3 replication in response to infection.

Our findings therefore reveal a novel mechanism by which CVB3 impairs autophagy flux, indicating that STX17 may be a potential target for antiviral therapy. These findings will also help to elucidate the detailed mechanisms of CVB3 infection in the future.

## Materials and methods

### Cell culture and transfection

HeLa cells were cultured in Dulbecco’s modified Eagle’s medium (DMEM; Invitrogen Life Technologies, Carlsbad, CA, USA) supplemented with 10% fetal bovine serum (Sigma-Aldrich Corp., St. Louis, MO, USA), and 50 μg/ml penicillin and streptomycin (regular medium) in an atmosphere comprising 5% CO_2_. The cells were transiently transfected using Lipofectamine 2000 reagent (Invitrogen Life Technologies, Carlsbad, CA, USA) with the plasmids Myc-STX17, mRFP-GFP-LC3, or siRNA, according to the manufacturer’s protocol.

### Virus infection

HeLa cells were contacted with CVB3 at an MOI of 10 (low MOI) or 50 (high MOI) for 1 h in serum-free DMEM to achieve infection. An equivalent amount of phosphate-buffered saline (PBS) was used for sham infection. The cells were then washed with PBS and cultured in complete medium for the indicated amounts of time until they were harvested.

### Immunofluorescence

Cells grown on coverslips were washed with PBS and fixed in 4% paraformaldehyde in PBS for 10 min at 4 °C. The fixed cells were permeabilized with 50 μg/mL digitonin in PBS for 5 min, blocked with 3% bovine serum albumin in PBS for 30 min, and incubated with an LC3-reactive antibody (#3868, Cell signaling Tech, Danvers, MA, USA) for 1 h. After washing, the cells were incubated with an Alexa Fluor 488-conjugated anti-rabbit IgG (Invitrogen Life Technologies, Carlsbad, CA, USA) for 30 min. Images were acquired under a confocal laser microscope (FV1000D IX81, Olympus, Corporation, Shinjuku-ku, Tokyo, Japan) using a 60× oil-immersion objective lens with a numerical aperture (NA) of 1.42 and 1.5× zoom, and captured using Fluoview software (Olympus, Corporation, Shinjuku-ku, Tokyo, Japan). The image size was set at 1024 × 1024 pixels.

### Immunoblotting

Cells were lysed with lysis buffer containing 1% Triton X-100, and the resulting samples separated by sodium dodecyl sulfate–polyacrylamide gel electrophoresis (SDS–PAGE) and transferred to Immobilon-P polyvinylidene difluoride membranes (Millipore Corporation, Billerica, MA, USA). Immunoblot analysis was performed and visualized using Super-Signal West Pico Chemiluminescent substrate (Pierce Chemical Co., Rockford, IL, USA) or Immobilon Western Chemiluminescent HRP substrate (Millipore Corporation, Billerica, MA, USA). Signal intensities were analyzed using an LAS-3000 mini imaging analyzer and Multi Gauge software, version 3.0 (Fujifilm, Tokyo, Japan).

### Measurement of cathepsin B/L activity

A cell-lysate-based assay of cathepsin B/L activity was performed using the ab65300 assay kit for cathepsin B, and the ab65306 assay kit for cathepsin L (both from Abcam Plc., Cambridge, MA, USA), according to the the manufacturer’s protocol. Briefly, cells were lysed in M2 buffer and the resulting lysate incubated with 50 μM fluorogenic cathepsin B/L substrate (Z-RR-AMC or Ac-HRYR-ACC, respectively) in a cell-free system comprising buffer (10 mM HEPES-NaOH, pH 7.4, 220 mM mannitol, 68 mM sucrose, 2 mM NaCl, 2.5 mM KH_2_PO_4_, 0.5 mM EGTA, 2 mM MgCl_2_, 5 mM pyruvate, 0.1 mM PMSF, and 1 mM dithiothreitol) in a 96-well plate for 1 h at 37 °C. The fluorescence intensity was monitored using a fluorometer (ThermoFisher Scientific Inc., Waltham, MA, USA) at an excitation wavelength of 400 nm and an emission wavelength of 505 nm.

The data represent relative fluorescence intensities compared with the control group.

### Detection of Triton X-100-soluble and Triton X-100-insoluble proteins

Cells were lysed on ice for 10 min in 0.1% Triton X-100 in TEM buffer (20 mM NaCl, 20 mM Tris-HCl, pH 7.4, 5 mM MgCl_2_, 0.1 mM EDTA), using a Dounce homogenizer, and the resulting total cell extracts were centrifuged at 16,000 × *g* for 15 min to separate the soluble (supernatant) and insoluble (pellet) fractions. Both fractions were submitted to SDS–PAGE and immunoblotting with an antibody against multiubiquitin (#3936, Cell signaling Tech, Danvers, MA, USA).

### Electron microscopy

HeLa cells were fixed in 2.5% glutaraldehyde in 0.1 M sodium phosphate buffer, pH 7.4, at 37 °C for 2 h, and then dehydrated in a graded ethanol series and embedded. Approximately 70-nm ultrathin sections were mounted on nickel grids. The samples were then stained and visualized using a 120-kV Jeol electron microscope (JEM-1400, JEOL Ltd, Akishima, Tokyo, Japan) at 80 kV. Images were captured using a Gatan-832 digital camera.

### Real-time PCR

Total RNA was extracted using the TRIzol reagent, according to the manufacturer’s instructions, digested with DNase (Invitrogen Life Technologies, Carlsbad, CA,USA), extracted with phenol–chloroform, and precipitated with ethanol to remove contaminating DNA. Total RNA (2.5 μg) from mock-infected and CVB3-infected cell samples was reverse-transcribed using random hexamers and SuperScript reverse transcriptase (Invitrogen Life Technologies, Carlsbad, CA, USA). The cDNA quantities were normalized to 18S rRNA. The following primers were used: STX17 forward, 5′-ATGTGTGTGGAGAGCGTCAA-3′ and reverse, 5′-ACAGTTC CACAAAGGCATCC-3′; Lamp1 forward, 5′-GAGCGCTTCAGTTCAGCTTT-3′ and reverse, 5′-CCCTTGAGCTGCAGACATTA-3′; Cyr61 forward, 5′-CACTAATGCAGC CACGATTG-3′ and reverse, 5′ -CATCAACTCCACAAGCTCCA-3′. Reactions were performed in 10-μL volumes that included a diluted cDNA sample, primers (0.5 μM), and LightCycler DNA Master SYBR green I mix (Roch LifeScience, Shanghai, China), which contained nucleotides, Taq DNA polymerase, and optimized buffer components. Real-time PCR reactions were performed on a LightCycler 2.0 detection system (Roch LifeScience, Shanghai, China). To compare mock-infected and infected cell samples, relative changes in gene expression were determined using the 2^−ΔΔCT^ method.

### Statistical analysis

Assays for characterizing cell phenotypes were analyzed using Student’s *t* test, and correlations between the groups were calculated using Pearson’s test. Differences with *p* < 0.01 were deemed statistically significant. Data were analyzed using GraphPad Prism 5 software (GraphPad Software, La Jolla, CA, USA).

## References

[1] Kearney MT, Cotton JM, Richardson PJ, Shah AM (2001). Viral myocarditis and dilated cardiomyopathy: mechanisms, manifestations, and management. Postgrad. Med. J..

[2] Kadambari S (2014). Enterovirus infections in England and Wales, 2000–2011: the impact of increased molecular diagnostics. Clin. Microbiol. Infect..

[3] Lee CJ (2014). Clinical features of coxsackievirus A4, B3 and B4 infections in children. PLoS ONE.

[4] Khetsuriani N, Lamonte A, Oberste MS, Pallansch M (2006). Neonatal enterovirus infections reported to the national enterovirus surveillance system in the United States, 1983–2003. Pediatr. Infect. Dis. J..

[5] Huber S, Ramsingh AI (2004). Coxsackievirus-induced pancreatitis. Viral Immunol..

[6] Tracy S (2000). Group B coxsackievirus myocarditis and pancreatitis: connection between viral virulence phenotypes in mice. J. Med. Virol..

[7] Gaaloul I (2012). Sudden unexpected death related to enterovirus myocarditis: histopathology, immunohistochemistry and molecular pathology diagnosis at post-mortem. BMC Infect. Dis..

[8] Alirezaei M, Flynn CT, Wood MR, Harkins S, Whitton JL (2015). Coxsackievirus can exploit LC3 in both autophagy-dependent and -independent manners in vivo. Autophagy.

[9] Kemball CC (2010). Coxsackievirus infection induces autophagy-like vesicles and megaphagosomes in pancreatic acinar cells in vivo. J. Virol..

[10] Tabor-Godwin JM, Tsueng G, Sayen MR, Gottlieb RA, Feuer R (2012). The role of autophagy during coxsackievirus infection of neural progenitor and stem cells. Autophagy.

[11] Levine B, Kroemer G (2008). Autophagy in the pathogenesis of disease. Cell.

[12] Shintani T, Klionsky DJ (2004). Autophagy in health and disease: a double-edged sword. Science.

[13] Levine B, Klionsky DJ (2004). Development by self-digestion: molecular mechanisms and biological functions of autophagy. Dev. Cell.

[14] Chiramel AI, Brady NR, Bartenschlager R (2013). Divergent roles of autophagy in virus infection. Cells.

[15] Shi J, Luo H (2012). Interplay between the cellular autophagy machinery and positive-stranded RNA viruses. Acta Biochim. Biophys. Sin. (Shanghai).

[16] Sir D, Ou JH (2010). Autophagy in viral replication and pathogenesis. Mol. Cell.

[17] Zhai X (2015). Coxsackievirus B3 induces autophagic response in cardiac myocytes in vivo. Biochemistry (Moscow)..

[18] Wong J (2008). Autophagosome supports coxsackievirus B3 replication in host cells. J. Virol..

[19] Madan V, Sanchez-Martinez S, Carrasco L, Nieva JL (2010). A peptide based on the pore-forming domain of pro-apoptotic poliovirus 2B viroporin targets mitochondria. Biochim. Biophys. Acta.

[20] Wu H (2016). Protein 2B of coxsackievirus B3 induces autophagy relying on its transmembrane hydrophobic sequences. Viruses.

[21] Kabeya Y (2000). LC3, a mammalian homologue of yeast Apg8p, is localized in autophagosome membranes after processing. EMBO J..

[22] Klionsky DJ (2016). Guidelines for the use and interpretation of assays for monitoring autophagy (3rd edition). Autophagy.

[23] Komatsu M (2007). Homeostatic levels of p62 control cytoplasmic inclusion body formation in autophagy-deficient mice. Cell.

[24] Tanida I, Ueno T, Kominami E (2008). LC3 and autophagy. Methods Mol. Biol..

[25] Kimura S, Noda T, Yoshimori T (2007). Dissection of the autophagosome maturation process by a novel reporter protein, tandem fluorescent-tagged LC3. Autophagy.

[26] Zhou J (2013). Activation of lysosomal function in the course of autophagy via mTORC1 suppression and autophagosome–lysosome fusion. Cell Res..

[27] Hara T (2006). Suppression of basal autophagy in neural cells causes neurodegenerative disease in mice. Nature.

[28] Xin L (2014). Coxsackievirus B3 induces crosstalk between autophagy and apoptosis to benefit its release after replicating in autophagosomes through a mechanism involving caspase cleavage of autophagy-related proteins. Infect. Genet. Evol..

[29] Yuan JP (2003). Coxsackievirus B3-induced apoptosis and caspase-3. Cell Res..

[30] Carthy CM (1998). Caspase activation and specific cleavage of substrates after coxsackievirus B3-induced cytopathic effect in HeLa cells. J. Virol..

[31] Henke A (2000). Apoptosis in coxsackievirus B3-caused diseases: interaction between the capsid protein VP2 and the proapoptotic protein siva. J. Virol..

[32] Xin L, Ma X, Xiao Z, Yao H, Liu Z (2015). Coxsackievirus B3 induces autophagy in HeLa cells via the AMPK/MEK/ERK and Ras/Raf/MEK/ERK signaling pathways. Infect. Genet. Evol..

[33] Alirezaei M, Flynn CT, Wood MR, Whitton JL (2012). Pancreatic acinar cell-specific autophagy disruption reduces coxsackievirus replication and pathogenesis in vivo. Cell Host Microbe.

[34] Tang B (2012). Compromised autophagy by MIR30B benefits the intracellular survival of *Helicobacter pylori*. Autophagy.

[35] Schmid D, Munz C (2007). Innate and adaptive immunity through autophagy. Immunity.

[36] Shi J (2013). Cleavage of sequestosome 1/p62 by an enteroviral protease results in disrupted selective autophagy and impaired NFKB signaling. Autophagy.

[37] Riley BE (2010). Ubiquitin accumulation in autophagy-deficient mice is dependent on the Nrf2-mediated stress response pathway: a potential role for protein aggregation in autophagic substrate selection. J. Cell Biol..

[38] Lennemann NJ, Coyne CB (2015). Catch me if you can: the link between autophagy and viruses. PLoS Pathog..

[39] Deretic V, Levine B (2009). Autophagy, immunity, and microbial adaptations. Cell Host Microbe.

[40] Itakura E, Kishi-Itakura C, Mizushima N (2012). The hairpin-type tail-anchored SNARE syntaxin 17 targets to autophagosomes for fusion with endosomes/lysosomes. Cell.

[41] Uematsu M, Nishimura T, Sakamaki Y, Yamamoto H, Mizushima N (2017). Accumulation of undegraded autophagosomes by expression of dominant-negative STX17 (syntaxin 17) mutants. Autophagy.

[42] Ding B (2014). Phosphoprotein of human parainfluenza virus type 3 blocks autophagosome–lysosome fusion to increase virus production. Cell Host Microbe.

[43] Ren H (2016). The autophagosomal SNARE protein syntaxin 17 is an essential factor for the hepatitis C virus life cycle. J. Virol..

[44] Lloyd RE (2006). Translational control by viral proteinases. Virus Res..

[45] Zaragoza C (2006). Viral protease cleavage of inhibitor of kappaBalpha triggers host cell apoptosis. Proc. Natl. Acad. Sci. USA.

[46] Yalamanchili P, Datta U, Dasgupta A (1997). Inhibition of host cell transcription by poliovirus: cleavage of transcription factor CREB by poliovirus-encoded protease 3Cpro. J. Virol..

[47] Saraste A (2003). Cardiomyocyte apoptosis in experimental coxsackievirus B3 myocarditis. Cardiovasc. Pathol..

[48] Yuan J (2005). Inhibition of glycogen synthase kinase 3beta suppresses coxsackievirus-induced cytopathic effect and apoptosis via stabilization of beta-catenin. Cell Death Differ..

[49] Kim SM (2004). Coxsackievirus B3 infection induces cyr61 activation via JNK to mediate cell death. J. Virol..

[50] Munch D (2014). Autophagy deficiency leads to accumulation of ubiquitinated proteins, ER stress, and cell death in *Arabidopsis*. Autophagy.

[51] Bensaad K, Cheung EC, Vousden KH (2009). Modulation of intracellular ROS levels by TIGAR controls autophagy. EMBO J..

[52] Wu JJ (2009). Mitochondrial dysfunction and oxidative stress mediate the physiological impairment induced by the disruption of autophagy. Aging (Albany, NY).

[53] Kyto V (2004). Glutathione depletion and cardiomyocyte apoptosis in viral myocarditis. Eur. J. Clin. Invest..

[54] Chen J, Noueiry A, Ahlquist P (2003). An alternate pathway for recruiting template RNA to the brome mosaic virus RNA replication complex. J. Virol..

[55] Welsch S, Locker JK (2010). Hijacking cellular garbage cans. Cell Host Microbe.

